# Differentially expressed lncRNAs and miRNAs with associated ceRNA networks in aged mice with postoperative cognitive dysfunction

**DOI:** 10.18632/oncotarget.18362

**Published:** 2017-06-03

**Authors:** Changwei Wei, Ting Luo, Shanshan Zou, Xiaobin Zhou, Wenzhen Shen, Xiaolin Ji, Qi Li, Anshi Wu

**Affiliations:** ^1^ Department of Anesthesiology, Beijing Chao-Yang Hospital, Capital Medical University, Beijing, China; ^2^ Department of Orthopedics, Beijing Chao-Yang Hospital, Capital Medical University, Beijing, China; ^3^ Department of Anesthesiology, Beijing Tsinghua Changgung Hospital, Tsinghua University, Beijing, China

**Keywords:** microarray, miRNA, long non-coding RNA, competing endogenous RNA, postoperative cognitive dysfunction, Gerotarget

## Abstract

Postoperative cognitive dysfunction (POCD) is a common postoperative complication observed in elderly patients. Using microarray analyses, we comprehensively compared long non-coding RNA (lncRNA), messenger RNA (mRNA), and microRNA (miRNA) expression profiles in hippocampal tissues from a mouse model of POCD and control mice. A total of 175 lncRNAs, 117 mRNAs, and 26 miRNAs were differentially expressed between POCD and control mice. Gene ontology (GO) and KEGG pathway enrichment analyses were performed to explore the principal functions of dysregulated genes. Correlated coding-noncoding co-expression (CNC) and competing endogenous RNA (ceRNA) expression networks were constructed using bioinformatics methods. lncRNA NONMMUT000708 correlated positively with expression of the inflammation-related gene *Hif3a*. lncRNAs NONMMUT043249 and NONMMUT028705 mediated gene expression by binding the transcription factor cAMP response element-binding protein (CREB). The constructed ceRNA network suggested lncRNA NONMMUT055714 binds competitively with miR-7684-5p, increasing expression of its target gene, *Sorl1*. Finally, eight dysregulated lncRNAs, four miRNAs, and ten mRNAs were confirmed *via* quantitative real-time polymerase chain reaction (PCR) in 10 POCD-healthy mouse paired samples. These results suggest that lncRNAs and miRNAs are involved in POCD pathogenesis and progression. Our ceRNA network will improve understanding of lncRNA-mediated ceRNA regulatory mechanisms operating during the pathogenesis of POCD.

## INTRODUCTION

Postoperative cognitive dysfunction (POCD) occurs following anesthetic operations and has numerous triggers. POCD is characterized by a lack of resilience to perioperative stress, which generates cognition-related clinical symptoms [1]. In 1998, the International Study of Postoperative Cognitive Dysfunction (ISPOCD) group conducted a multi-center, systematic neuropsychological assessment using six individual tests to examine POCD incidence among elderly patients who underwent major abdominal, non-cardiac thoracic, or orthopedic surgery [2]. POCD was present in 25.8% of patients seven days post-surgery, and in 9.9% of the patients three months after surgery. This number was reduced to 1% 12 months following the surgery. The specific mechanisms underlying POCD remain unclear, although these likely include β-amyloid (Aβ) deposition [3], phosphorylation of tau proteins [4], inflammation [5–7], and neuronal apoptosis [8, 9]. POCD often leads to delayed post-operative recovery as well as prolonged hospital stays, and increases medical expenses. The molecular mechanisms underlying POCD must therefore be elucidated to develop targeted drugs for prevention, diagnosis, and treatment.

MicroRNAs (miRNAs) are small, single-stranded, non-coding RNAs with approximately 22 nucleotides, which mediate homologous sequence-dependent gene silencing in cells. The brain has the greatest number of enriched miRNAs, which are expressed in a developmental stage-specific, tissue-specific, and cell-specific manner [10]. miRNAs play important roles in development of the neural system, learning, and memory, and cause numerous neurological diseases [11–14]. Long non-coding RNAs (lncRNAs) are RNAs with transcript lengths of > 200 nucleotides, and regulate gene expression through multiple mechanisms. In 2011, Salmena, *et al*. presented the competing endogenous RNA (ceRNA) hypothesis [15]. In addition to known miRNAs, which affect target RNA stability at the post-transcriptional level, RNAs can, in turn, influence miRNAs by binding target genes. Through miRNA response elements (MREs), various types of RNA transcripts compete with one another for binding to a common miRNA, thereby regulating miRNA-mediated gene silencing. As ceRNAs, lncRNAs have been implicated in the progression of multiple diseases [16–18]. However, the potential roles of lncRNAs as ceRNAs in POCD remain unexplored.

The present study was designed to elucidate the molecular mechanisms underlying non-coding RNAs in POCD. Microarray analysis was performed to compare the hippocampal tissues of mice with POCD (the POCD group) and healthy mice (the control group), and to identify differentially expressed lncRNAs, mRNAs, and miRNAs. We employed GO and KEGG Pathway enrichment analyses to examine differentially expressed genes, and constructed a novel co-expression network of coding-noncoding genes and ceRNAs. Through analysis of the lncRNA/mRNA/miRNA co-expression network, we predicted roles for lncRNAs as ceRNAs in POCD occurrence and development. Finally, we used fluorescence quantitative real-time PCR (qRT-PCR) to confirm expression of 22 genes associated with POCD. Our findings provide a foundation for future research on the potential roles of lncRNAs and miRNAs in POCD.

## RESULTS

### Behavioral comparisons between POCD and control mice

**Figure 1:**
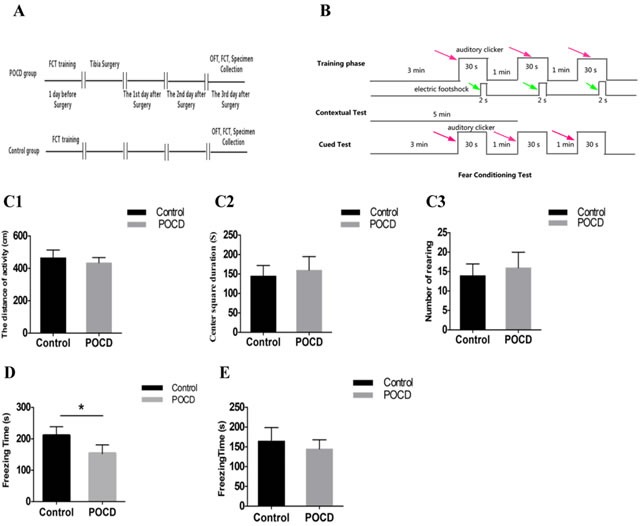
Experimental design, FCT workflow, and behavioral test results Two groups of mice were given behavioral training 1 d prior to orthopedic surgery (the control group received no specific treatment). Behavioral tests were performed on the third day post-surgery **A.** FCT: fear conditioning test. OFT: open field test. Red arrows indicate sound stimuli and green arrows indicate electric shock stimuli **B.** The two groups were subjected to behavioral training 1 d prior to surgery (training phase) and behavioral tests were performed on the third day post-surgery (contextual and cued tests). In the OFT, no noticeable difference was detected in general locomotion activity **C1.**, center square duration **C2.**, or rearing **C3.** between the two groups. Freezing time was lower in POCD mice than control mice in the contextual test **D.**, but was not different in the cued test **E.** *P < 0.05.

The behavioral testing design is shown in Figure 1A. The fear conditioning test (FCT) workflow is described in Figure 1B. No differences were detected between control and POCD mice in terms of general locomotion activity, center square duration, or rearing in the OFT (Figure 1C). In the contextual test, the average freezing time of POCD group mice was shorter than that of control mice (*P* < 0.05) (Figure 1D). However, there was no difference between POCD and control group freezing times in the cued test (Figure 1E).

### Differentially expressed lncRNAs, mRNAs, and miRNAs

**Figure 2:**
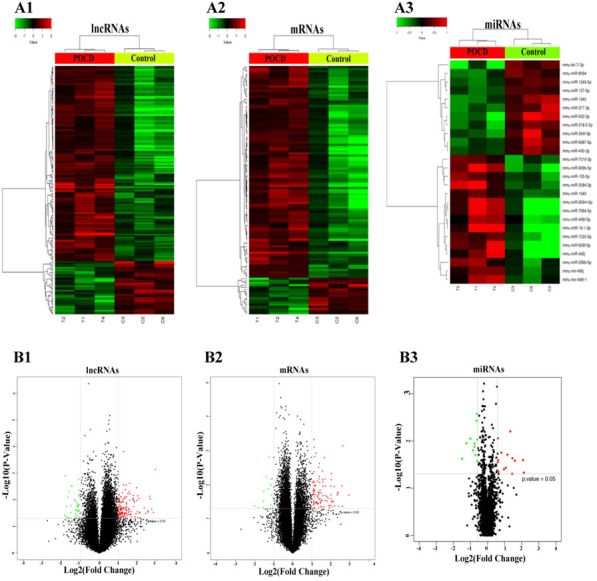
Heatmap and volcano plots showing lncRNA, mRNA, and miRNA levels Screening criteria were: FC > 2.0 and P ≤ 0.05 for lncRNAs **A1.** and mRNAs **A2.**, and FC > 1.5 and P ≤ 0.05 for miRNAs **A3.** Expression values are depicted in line with the color scale; intensity increases from green to red. Each column represents one sample, and each row indicates a transcript. Volcano plots reflecting number, significance, and reliability of differentially expressed lncRNAs **B1.**, mRNAs **B2.**, and miRNAs **B3.** The abscissa is log2 (FC value) and the ordinate is -log10 (P-value). Red dots are upregulated genes, green dots are downregulated genes, and black dots are genes that were the same between the two groups.

Microarray analysis was used to identify differentially expressed genes in the hippocampal tissues of three pairs of mice (Figure 2A). lncRNA and mRNA screening criteria were: fold change (FC) > 2.0 and *P* ≤ 5.0. miRNA screening criteria were: FC > 1.5 and *P* ≤ 0.05. A total of 175 differentially expressed lncRNAs were identified using microarray analysis, among which, 138 were upregulated and 37 were downregulated. The FC values for upregulated lncRNAs Gm28633 and NONMMUT060658 were > 5 (*P* < 0.01). 117 mRNAs were differentially expressed; 100 were upregulated and 17 were downregulated. 26 miRNAs were differentially expressed; 15 were upregulated and 11 were downregulated. Upregulated miR-466j exhibited an FC value > 4. All original data have been uploaded and are available at GEO. The miRNA microarray raw data number is GSE95070 and the lncRNA and mRNA raw data number is GSE95426. Using the P-value of the paired sample *t*-test and the FC values, a volcano plot was constructed to demonstrate differences between the two groups (Figure 2B).

### GO and KEGG pathway analysis

**Figure 3:**
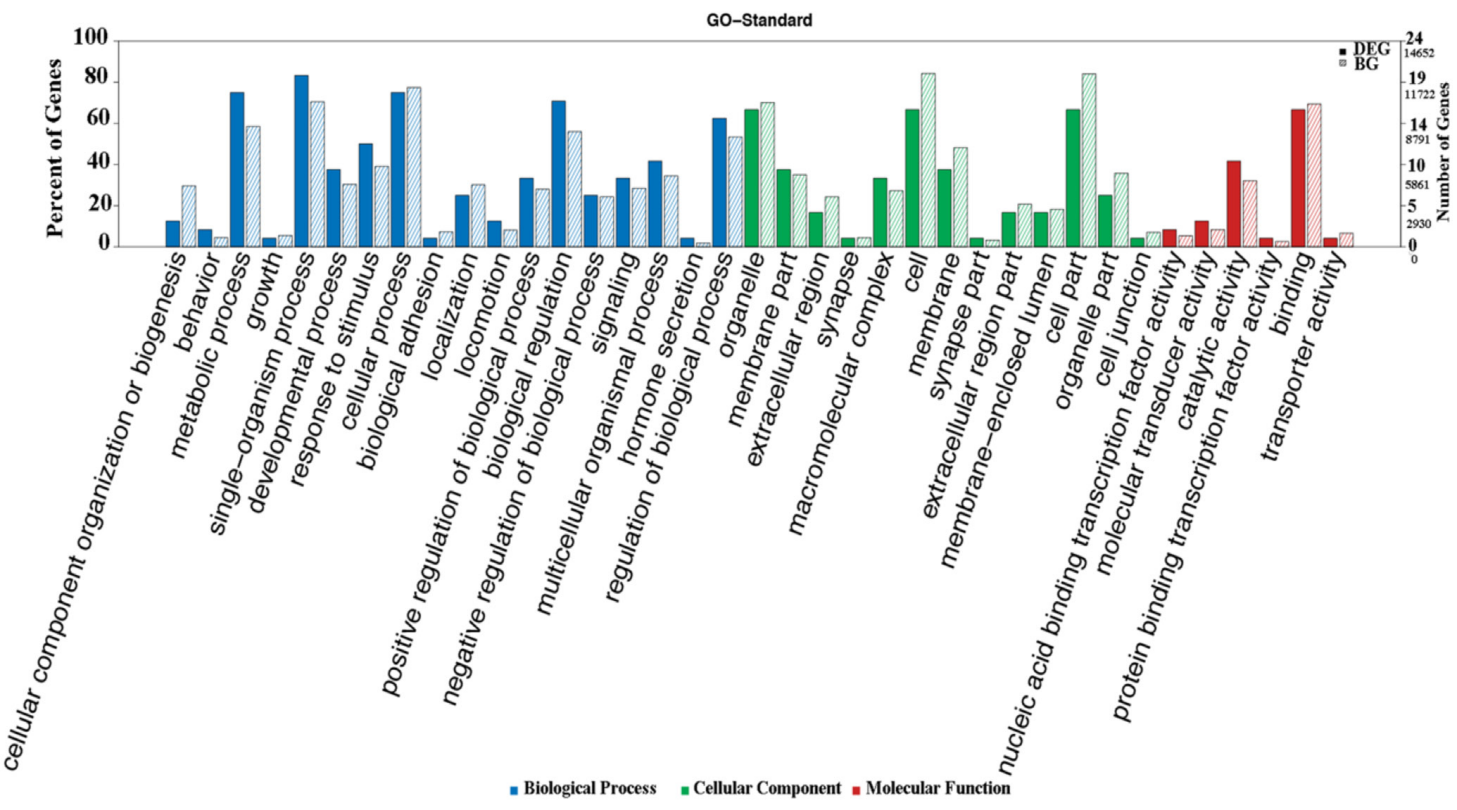
GO secondary ID frequency analysis The abscissa of the chart is the classification of the GO database. The left ordinate is the percent of genes. Solid bars: ratio of the number of differentially expressed genes enriched to a secondary function of the GO database to all differentially expressed genes; grid bars: ratio of the number of background genes enriched to a secondary function of the GO database to all genes. The percentage of differentially expressed genes enriched to a secondary function of the GO database may be higher or lower than the percentage of the background genes. The right ordinate is the number of genes. Each node on the right ordinate includes two numbers; the smaller is the number of differentially expressed genes and the larger is the number of background genes. Blue bars are biological processes, green are cellular components, and red are molecular functions.

Figure 3 shows gene enrichment in the context of differentially expressed genes between the two groups and in all genetic backgrounds. If the number or frequency of differentially expressed genes is different from the number or frequency of background genes in GO database entries, the differentially expressed genes may be related to a secondary function in the GO database. A GO enrichment analysis on the differentially expressed mRNAs showed that they were mainly related to biological processes and cellular components, and only a few differentially expressed genes were associated with molecular function.

**Figure 4:**
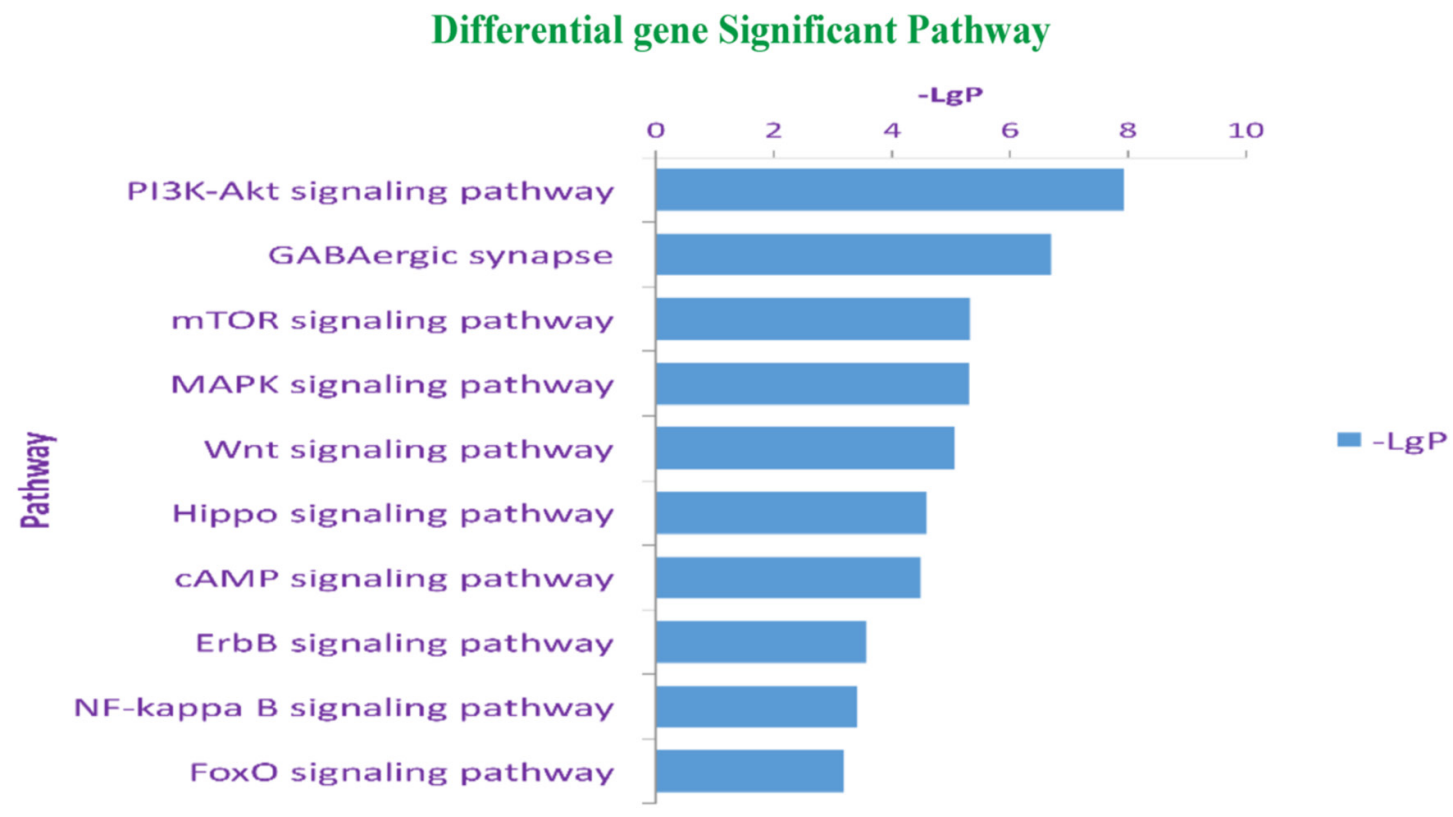
KEGG pathway enrichment analysis for mRNAs with the ten highest enrichment scores Pathway enrichment analysis includes two steps: (1) map input gene sets into the database, and then annotate the pathways involved in these gene sets; (2) compare the results obtained in the previous step with the background genes, and identify significant enrichment pathways. Statistical testing was performed using Hypergeometric Distribution. The abscissa is -Log P-value (-LgP). The bigger the -LgP, the smaller the P-value, indicating that enrichment of differentially expressed genes in a given pathway was significant.

KEGG pathway enrichment analysis targeting differentially expressed mRNAs (the 10 pathways with the highest enrichment scores were selected) revealed that differentially expressed mRNAs were mainly involved in Wnt, PI3K-AKT, and mTOR signaling. Based on pathway enrichment analysis results, we speculated that pathways enriched with differentially expressed genes might be involved in POCD occurrence and development (Figure 4).

### lncRNA/mRNA co-expression and function prediction

**Figure 5:**
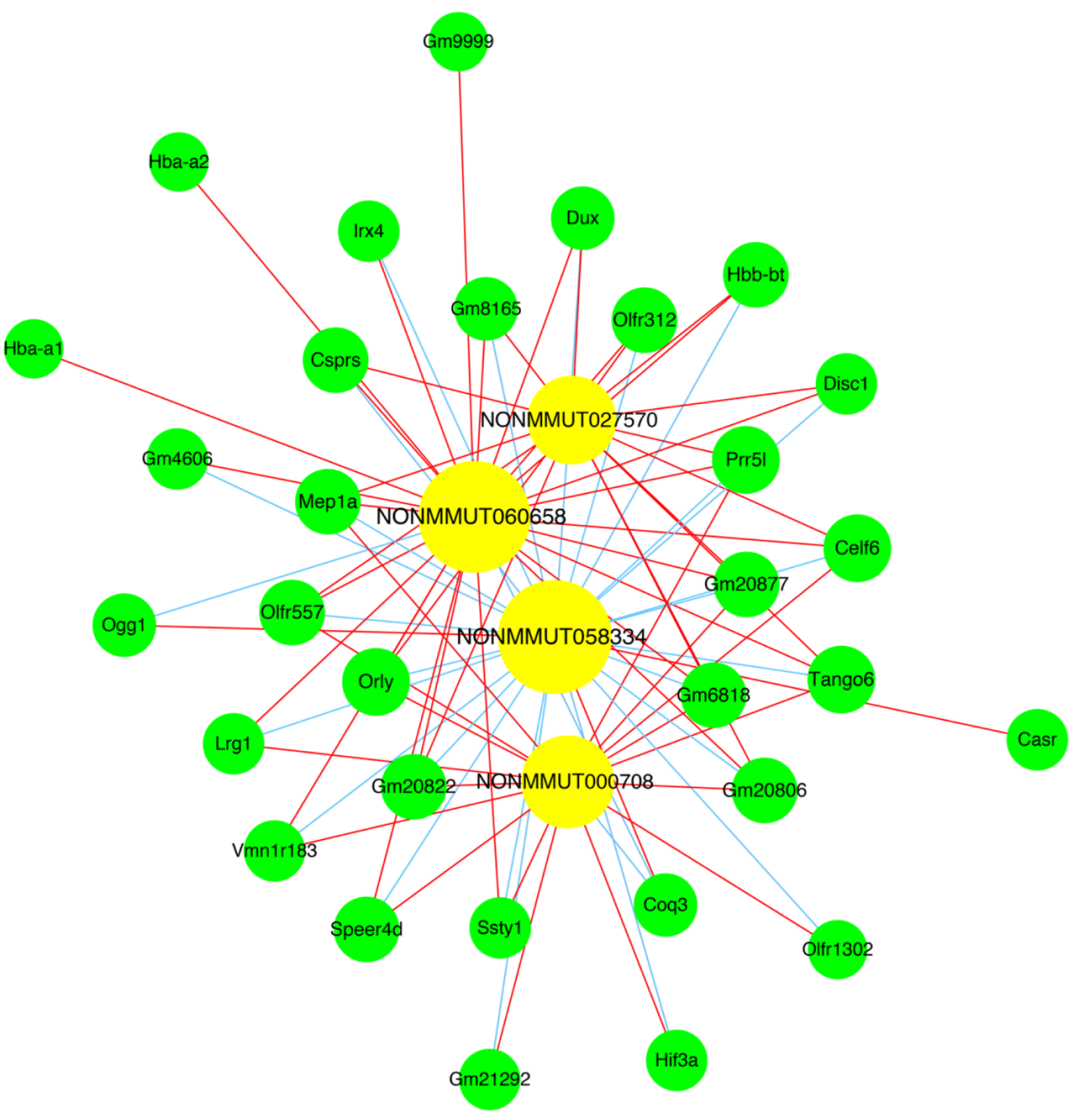
Co-expression network of four lncRNAs with associated mRNAs Co-expressed lncRNA-mRNA pairs were identified using strict screening criteria (correlation coefficients > 0.90 or < -0.90, P < 0.01). Yellow circles represent lncRNAs, green circles represent mRNAs, red lines show positive correlations, and blue lines show negative correlations.

At present, lncRNA function predictions are mainly based on their co-expression with coding genes. After a strict screening process, co-expressed lncRNA-mRNA gene pairs (correlation coefficient > 0.90 or < -0.90, and *P* < 0.01) were selected (Figure 5). lncRNA NONMMUT060658 was positively correlated with *Prr5l, Irx4, Celf6, Lrg1*, and *Speer4d* levels, and negatively correlated with *Coq3* expression. lncRNA NONMMUT058334 was negatively correlated with *Orly*, *Dux, Speer4d*, and *Tango6* levels. lncRNA NONMMUT000708 was positively correlated with *Hif3a* expression. Co-expressed mRNAs were involved in inflammation, neuronal apoptosis, and metabolic pathways. We established a coding-noncoding gene expression network to analyze non-coding gene functions.

### Predicting lncRNA target genes and binding with transcription factors

Based on lncRNA and mRNA co-expression patterns (correlation coefficients > 0.90 or < -0.90, and *P* < 0.01), we predicted potential targets of lncRNAs in *cis* (cis-prediction) and *trans* (trans-prediction). Cis-prediction identified lncRNA-mRNA pairs located within 10 kb of one another, while trans-prediction screened lncRNA-mRNA pairs for sequence similarities using the BLAT algorithm (default parameter setting) to align lncRNA and mRNA sequences (3′-UTR). Our findings indicated that NONMMUT034640 regulates *Zfp459* and *Celf6* expression in *trans*.

For each lncRNA, we predicted transcription factor binding 2000 bp upstream to 500 bp downstream of the lncRNA initiation site. lncRNAs, NONMMUT043249, NONMMUT028705, XR_886465.1, and PX00200H22 can bind cAMP response element-binding protein (CREB). lncRNA XR_377638.2 can bind with the signal transducer and activator of transcription 3 (STAT3). NONMMUT060658 can bind transcription factors, FOXD3 and HNF-1, and NONMMUT000984 can bind Evi-1 and Oct-1.

### Construction of the ceRNA network

**Figure 6:**
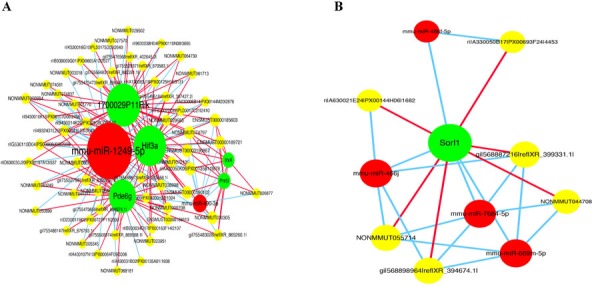
Competing endogenous RNA network in POCD mice The competing endogenous RNA network is based on miRNA-mRNA, miRNA-lncRNA, and lncRNA-mRNA interactions. Red circles represent miRNAs, yellow circles represent lncRNAs, and green circles represent mRNAs. The link in red indicates a positive correlation between the two connected nodes; the link in blue indicates a negative correlation.

We constructed the ceRNA network based on co-expressed miRNAs-mRNAs, miRNAs-lncRNAs, and lncRNAs-mRNAs (Figure 6A-6B). lncRNA NONMMUT060658 can act as a ceRNA and competitively binds miRNA-1249-5p, increasing *Hif3a* expression. lncRNA NONMMUT055714 binds competitively with miR-7684-5p, increasing *Sorl1* expression.

### Validation of deregulated lncRNAs, mRNAs, and miRNAs

**Figure 7:**
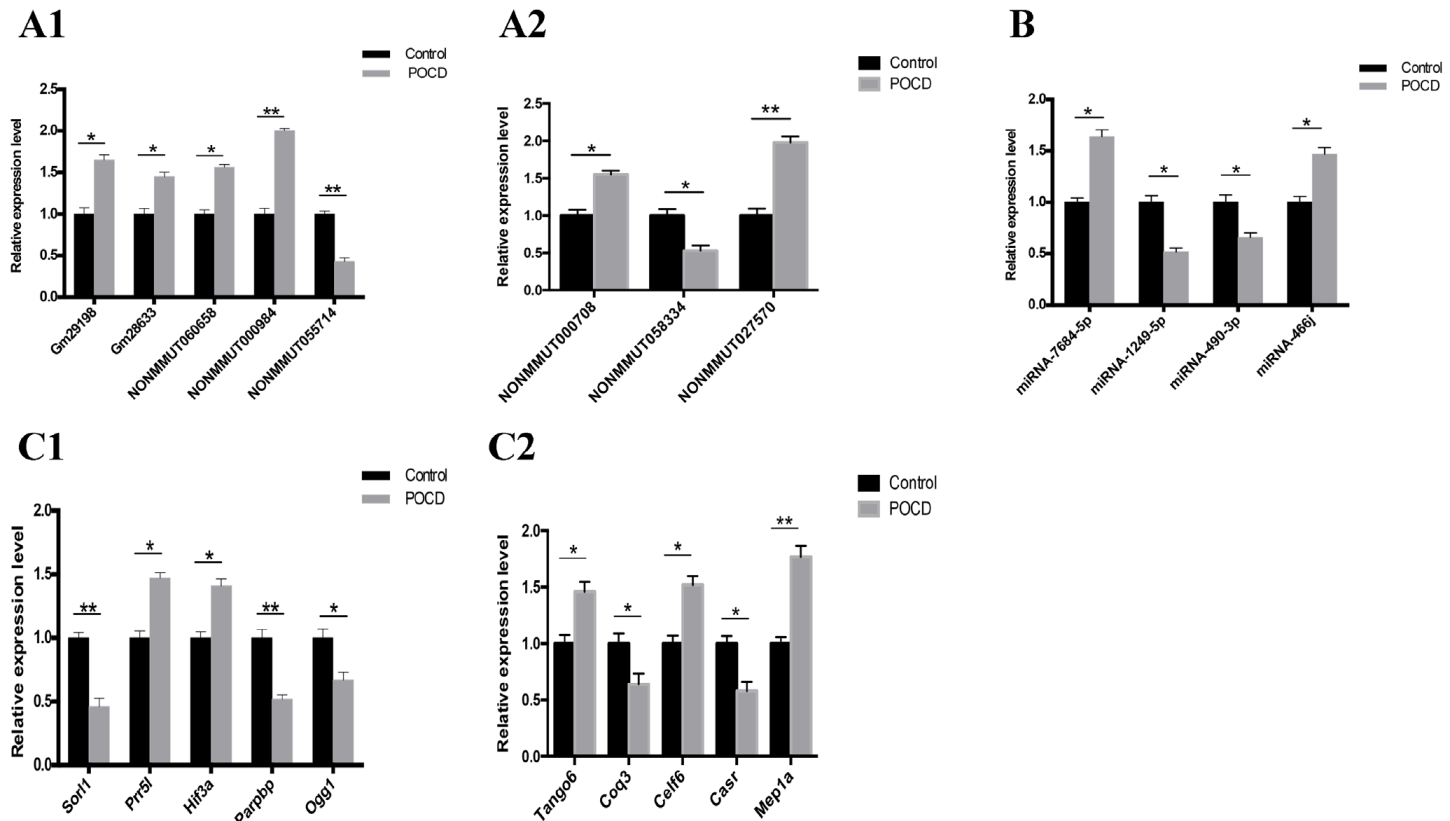
Transcript validation *via* quantitative RT-PCR Relative expression of eight lncRNAs **A.**, four miRNAs **B.**, and ten mRNAs **C.** between the POCD and control groups are shown. *P < 0.05, **P < 0.01.

Based on bioinformatic prediction results, fold changes ( > 2.0), and P-values (*P* < 0.05), we selected eight lncRNAs, four miRNAs, and ten mRNAs identified from the hippocampal tissues of 10 pairs of mice to validate *via* qRT-PCR analysis. lncRNAs Gm29198, Gm28633, NONMMUT060658, NONMMUT000984, NONMMUT000708, and NONMMUT027570 were upregulated, while NONMMUT055714 and NONMMUT058334 were downregulated (Figure 7A). miR-7684-5p and miR-466j were upregulated, while miR-1249-5p and miR-490-3p were downregulated (Figure 7B). *Prr5l*, *Hif3a*, *Tango6*, *Celf6*, and *Mep1a* mRNAs were upregulated, while *Ogg1, Sorl1*, *Parpbp, Coq3,* and *Casr* were downregulated (Figure 7C). qRT-PCR analysis results were consistent with those of the microarray analyses, confirming the reliability of the microarray results.

### Construction of the Circos plot

**Figure 8:**
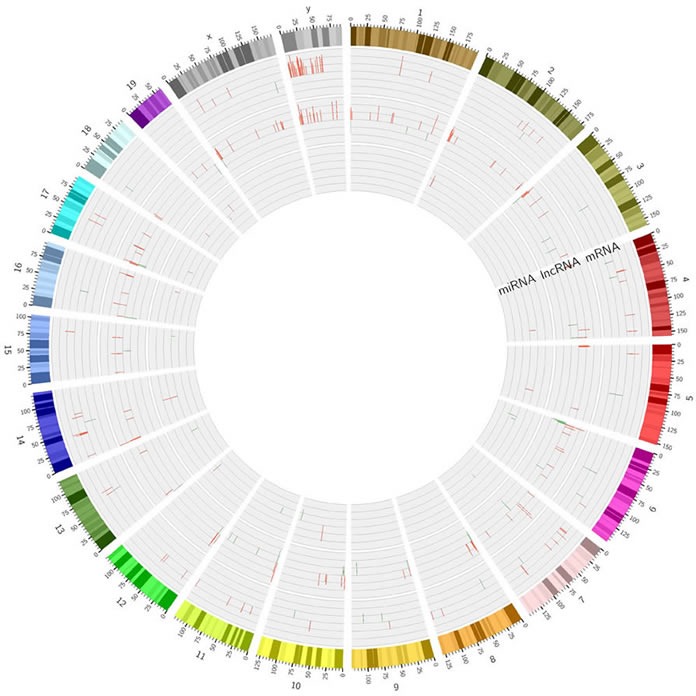
Differential expression of miRNAs, lncRNAs, and mRNAs Upregulation is indicated in red, downregulation is shown in green. Height indicates degree of difference in expression.

A Circos plot was constructed using the Circos software package to demonstrate miRNA, lncRNA, and mRNA differential expression (from the inner to outer layer) at the genomic level (Figure 8). Upregulation is shown in red and downregulation is shown in green. Height indicates the degree of difference in gene expression.

## DISCUSSION

In clinical practice, POCD incidence is higher in patients undergoing orthopedic surgery, especially among elderly patients. The present study employed the animal model of POCD for orthopedic surgery developed by Terrando, *et al*. [19]. On the third day post-surgery, POCD and control mice showed no differences in general locomotion activity, center square duration, or rearing in the OFT, suggesting that no motor dysfunction was detected in mice after orthopedic surgery. The two groups were then subjected to FCT using the contextual and cued tests to assess the abilities of mice to learn an association between environmental cues and aversive experiences. Previous studies in animals showed that the hippocampus is critical in FCT, and FCT assessment has become a common method for investigating hippocampal-dependent associative memory in models of POCD [20]. The major brain areas involved in contextual and cued fear conditioning include the amygdala, hippocampus, frontal cortex, and cingulate cortex. The frontal/cingulate cortices are areas of attentional learning and are involved in acquisition of new memories [21, 22]. Freezing time, a state of immobility that small rodents tend to present when faced with fear, was shorter in the POCD group than in the control group in the contextual test, indicating that POCD group mice had learning and memory dysfunctions following orthopedic surgery.

On the third day post-surgery, hippocampal tissues were collected from both groups to conduct a microarray screening to identify differentially expressed genes. We identified 175 differentially expressed lncRNAs, 117 differentially expressed mRNAs, and 26 miRNAs. Some of the differentially expressed genes were involved in several essential *in vivo* biological processes. Prior studies showed that miR-490-3p regulates cell proliferation and apoptosis by targeting *CCND1, CDK1*, and *HMGA2* [23–25]. Lrg1 promotes angiogenesis by modulating transforming growth factor-β (TGF-β) signaling [26]. Ogg1 is involved in immune inflammation, homeostasis, macrophage activation, and regulation of liquid-surface tension through mediation of chemokines, cytokines, integrins, and interleukin signaling [27, 28]. Sorl1 regulates the amyloid precursor protein (APP) metabolic pathway. When newly synthesized APP passes through the Golgi to the plasma membrane, some APP is deconstructed into sAPPα, a non-processed precursor endocytosed from the cell surface into late endosomal compartments for processing into sAPPβ and Aβ. As a sorting protein receptor, Sorl1 can trap APP in the Golgi. Sorl1 can also shuttle APP in the early endosome and the Golgi, upregulating pathways that form soluble APP, downregulating those that form insoluble amyloid, and inhibiting Aβ production and its accumulation in the brain [29]. Thus, Sorl1 is considered the key factor in the pathogenesis of Alzheimer’s disease (AD).

While the specific mechanisms underlying POCD remain unclear, we speculated that the differentially expressed genes identified in our study might promote POCD. Based on GO enrichment analysis results, differentially expressed mRNAs were mainly associated with biological processes and cellular components, and only a small proportion of differentially expressed genes was associated with molecular function. Differentially expressed mRNAs were mainly enriched in biological regulation and metabolic processes in the biological process category.

KEGG pathway analysis for the differentially expressed mRNAs revealed 10 pathways that could be implicated in POCD, including Wnt, PI3K-AKT, phosphatidylinositol, mTOR, and MAPK signaling. These pathways are known to be involved in occurrence and development of neurodegenerative diseases. PI3K-AKT signaling mediates isoflurane anesthesia or surgery-induced inflammation, oxidative stress, and neuronal cell apoptosis, leading to cognitive impairment [30]. We previously showed that activation of mTOR signaling increased β-amyloid production and tau protein phosphorylation, leading to decreased cognitive function after surgery [31]. Wnt signaling mediates synaptic efficacy in the neural circuits of the central nervous system. Intracellular accumulation of β-catenin is a typical Wnt pathway activation marker, and β-catenin tends to be reduced in AD patients [32]. Hence, promoting β-catenin synthesis can enhance expression of Wnt target genes, thereby inhibiting neuronal apoptosis.

To date, the functions of most lncRNAs are unknown. Establishment of a co-expression network of ceRNAs can be used to predict lncRNA function, as these functions can be deduced from the coding genes in the co-expression network. In our study, lncRNA NONMMUT060658 expression correlated positively with that of *Prr5l, Irx4, Celf6*, *Lrg1*, and *Speer4d*, and negatively with that of *Coq3*. lncRNA NONMMUT058334 expression correlated negatively with that of *Orly, Dux, Speer4d*, and *Tango6*, and lncRNA NONMMUT000708 expression correlated positively with that of *Hif3a*. The mRNAs in the co-expression network were involved in inflammation, neuronal apoptosis, and metabolic pathways [33–35]. Our findings suggest that these differentially expressed lncRNAs participate in POCD by interacting with their corresponding coding genes.

Some lncRNAs can act as ligands and bind transcription factors to form complexes that control target gene transcriptional activity. In our study, lncRNAs NONMMUT043249, NONMMUT028705, XR_886465.1, and PX00200H22 could bind CREB, and lncRNA XR_377638.2 could bind STAT3. CREB functions in transcriptional regulation through phosphorylation. It is a key regulator of long-term memory formation and can facilitate long-term potentiation (LTP) in animals, including mice [36, 37]. In addition, changes in CREB phosphorylation can affect neuronal synaptic plasticity and the formation of neural networks [38]. STAT3 is a signal transducer and transcriptional activator, transmitting extracellular signals into the nucleus. It participates in many physiological processes, including cellular growth, proliferation, differentiation, and apoptosis, as well as the development of the central nervous system. STAT3 plays an important role in learning and memory in both humans and animals, and is closely associated with cognitive dysfunction and dementia [39]. Thus, these differentially expressed lncRNAs could promote POCD progression by binding transcription factors.

The ceRNA hypothesis states that mRNA, lncRNA, pseudogene transcripts, and circular RNAs (circRNAs) compete to bind the same miRNA to influence target gene stability or translational activity, thereby regulating gene expression at the post-transcriptional level. In this study, we constructed a ceRNA network based on miRNA-mRNA, miRNA-lncRNA, and lncRNA-mRNA co-expression patterns. For example, as a ceRNA, lncRNA NONMMUT055714 competes for binding to miR-7684-5p, thereby affecting *Sorl1* expression. *Sorl1* increases Aβ production in the brains of elderly patients, increasing AD risk [29]. Additionally, Aβ over-deposition can trigger oxidative stress and autophagy, induce neuronal apoptosis and pro-inflammatory cytokine-dependent activation of glial cells, cause synaptic dysfunction, and accelerate abnormal phosphorylation of tau proteins, thus promoting POCD [40, 41]. Further research on NONMMUT055714 as a competitive endogenous RNA regulating *Sorl1* expression in postoperative cognitive dysfunction is underway in our laboratory. Understanding this novel RNA crosstalk will provide insights into gene regulatory networks with implications in human development and diseases.

Our study presents some limitations. First, we only screened genes differentially expressed in POCD mice on the third day after surgery. Thus, we do not know whether gene expression changed with time post-surgery. Second, while our bioinformatics analyses found that some of the differentially expressed genes may be related to POCD, these genes must be validated in future studies. Finally, we screened differentially expressed genes in a small number of samples. In our study protocol, five pairs of hippocampal tissues were used for microarray screening, but two pairs could not be included because of poor clustering. This can be explained two possible ways. First, although we randomly selected mice from the two groups, we cannot avoid biological differences between individuals. Second, RNA degradation may have occurred during the process of hippocampal tissue collection or total RNA extraction, which led to the poor clustering between the two pairs of tissue. However, all behavioral test results were performed on eight mice in each group. We used the two tailed test; effect size was 1.523 (calculated by the mean and standard deviation of the two groups), alpha = 0.05, and Power (1- beta) was 0.81 as calculated using G*Power software.

In conclusion, we employed microarray analyses to screen differentially expressed lncRNAs, miRNAs, and miRNAs in hippocampal tissues from POCD and control mice. Through GO and KEGG pathway enrichment analyses and construction of a co-expression network of lncRNAs-mRNAs and ceRNAs, we assessed the functions of these differentially expressed genes, correlated pathways, and mutual regulatory relationships between coding and noncoding genes. Our findings contribute to the understanding of POCD pathogenesis and provide a foundation for future studies of the molecular mechanisms underlying POCD.

## MATERIALS AND METHODS

### Animals

C57BL/6 mice used in this study were purchased from Beijing Vital River Laboratory Animal Technology Co., Ltd. (Beijing, China). Mice were male, aged 12-14 months, and weighed 25-35 g. Room temperature and humidity were controlled at 25°C and 55%, respectively. All mice were acclimated to the environment for one week prior to study initiation. The experiment was conducted in accordance with the “Guide for the Care and Use of Laboratory Animals” issued by the National Institutes of Health, and was approved by the Animal Ethics Committee of the Beijing Chao-Yang Hospital, Capital Medical University. Mice were randomly assigned into two groups: the POCD group and the control group (*n* = 8 mice per group). Both control and POCD group mice were given behavioral training (pre-operation training for the Fear Conditioning Test [FCT]) one day prior to POCD group surgery, and two behavioral tests (Open Field Test [OFT] and FCT) on the third day following surgery. Mice were anesthetized using isoflurane; hippocampal tissue was removed, immediately placed in liquid nitrogen, and then stored at −80°C until further examination.

### Animal model of POCD

The surgical model [19] can be described as follows: A left tibial fracture was performed with an intramedullary nail fixation on the mice from the POCD group under isoflurane anesthesia (2% isoflurane for induction, and 1.5% maintenance; Baxter International, Inc., Deerfield, IL, USA) and butorphanol (0.1 mg/kg, subcutaneous injection; Jiangsu Hengrui Medicine Co. Ltd., Lianyungang, China). Mouse left hind paws were disinfected three times with povidone-iodine, and scalpel was used to perform a longitudinal incision on the paw. Following the incision, a 0.38 mm pin was inserted in the tibial medullary canal. Following stripping of the periosteum, osteotomy, and irrigation, the skin of the wound was closed with 5-0 nylon sutures. Isoflurane was discontinued immediately after the operation, and animals were placed back in their cages, where they awoke naturally. After the operation, a heating pad and a temperature control lamp were used to maintain mouse body temperatures at approximately 37°C. Behavioral tests (OFT and FCT) were performed on the third day after the operation. Control mice were assessed using the same behavioral tests at the same time as POCD group mice, but did not receive any prior treatment or surgery.

### Open field test (OFT)

OFT [42] was used to evaluate anxiety and locomotor activity in experimental animals. A mouse was placed directly into the center of the open field (100 cm × 100 cm × 48 cm, length × width × height). Mouse movements were recorded using a digital camera during 5-min testing sessions. General locomotion activity (total number of grid lines crossed by each mouse), center square duration (time spent by each mouse in the central square), and rearing (frequency at which each mouse stands on their hind legs in the open field) were counted.

### Fear conditioning test (FCT)

The purpose of the FCT is to investigate the animals’ abilities to learn and memorize associations between unpleasant experiences and environmental implications. Sound and light are commonly used as conditioning stimuli (CS), while an aversive stimulus (such as an electric shock to the foot) acts as an unconditional stimulus (US). The two types of stimuli appear in pairs during the test. After CS-US training, in addition to the association between the sound and electric shock, animals are able to associate the link between the electric shock and the surrounding environment.

One day prior to the operation, after a three-min exploration period, mice were given three pairs of sound stimuli (2000 Hz, 90 Db, and 30 sec) and electric shock stimuli (1 mA, 2 sec). Each electric shock stimulus was given at the last two sec of the corresponding sound stimulus, and both ended at the same time. There was an interval of one min between two pairs of stimuli.

On the third day following the operation, the contextual test and the cued test were performed. During the contextual test, mice were placed in a context similar to that of the pre-operation training for a five-min observation period without the stimulation of sound and electric shock. During the cued test, two h after the completion of the contextual test, mice were placed in a context different from that of the pre-operation training (the interior was changed). After a three-min exploration period, mice were given three sound stimuli (2000 Hz, 90 Db, and 30 sec) without an electric shock stimulus. The interval between two stimuli was one min. The observation time lasted five min. Image analysis software was utilized to calculate the freezing times of the mice in the contextual and cued tests.

### RNA extraction

Total RNA was extracted from the POCD and control group hippocampal tissues using TRIzol (Invitrogen, Carlsbad, CA, USA) according to the manufacturer’s instructions. Total RNA was assessed *via* electrophoresis on a denaturing agarose gel and quantified using a NanoDrop spectrophotometer (NanoDrop, Wilmington, DE, USA).

### Quantitative real-time PCR validation

Ten pairs of hippocampal tissues were used for qRT-PCR validation. After RNA isolation, M-MLV reverse transcription (Promega, Madison, WI, USA) was used to synthesize cDNA. Quantitative PCR analysis and data collection were performed on the ABI 7500 qPCR system (Applied Biosystems, Foster City, CA, USA). Primer pairs are listed in Table 1. Relative gene expression was calculated using the 2^−ΔΔCt^ method, and fold changes are shown as means ± standard deviation (SD) from three independent experiments. U6 and β-actin were used as references.

**Table 1:** PCR primers used in this study

	Forward	Reverse
Gm29198	5′TCCAGTCATACCAGGGCTTC3′	5′GGCTTTGTTGCCCAATAGAC3′
Gm28633	5′GATCCGGCACCACATTTACT3 ‘	5′CCTTGCTGCTCTGGAGACTT3
NONMMUT060658	5′TTCCACCTGGTCAGAGATCC3′	5′GATGCCTCTGCAAATTGGTT3′
NONMMUT055714	5′CAGCTGGCATAGGGTGTGTA3′	5AAAGAGGCAATCAATGAAGAGC3′
NONMMUT000984	5′CAGTGTCAGCAGGTGGAAGA3′	5TAAGGCCTGGAATCCTGATG3′
NONMMUT000708	5′CAGCTCCTGCTTTCTGACCT3′	5′CCTGCACAAACATCATGACC3′
NONMMUT058334	5′GCATCTGAAACGCAGAACCT3′	5AGCGCAGAGGTCAGTGAAAT3′
NONMMUT027570	5′AAAGAGGCACCAGTTTCACG3′	5CTGCTTGCTGGCTATTCCTC3′
miR-7684-5p*	5′TCTGGGAAGCCTGGGCAGCA3′	
miR-1249-5p*	5′ATAGGAGGGAGGGGATGGGC3′	
miR-490-3p*	5′CAACCTGGAGGACTCCATGCTG3′	
miR-466j*	5′CGTGTGTGCATGTGCATGTGTGTAA3′	
*Ogg1*	5′CCCTAGAGGAGCTGGAAACC3′	5′CAGCAGTCTCACACCTTGGA3
*Sorl1*	5′CAGCACCTTGACCTGTACGA3′	5′GCCTTCTCATCGCTCTCATC3′
*Prr5l*	5′TCTGGGACCACTTCTTCACC3′	5′GCTTCACCTTCAGCAAGACC3′
*Hif3a*	5′GCAATGCCTGGTGCTTATCT3′	5′TCCTCTCGTCGCAGTATGTG3′
*Parpbp*	5′CACATGCCAGAGTCACCAGT3′	5′TTGAGAATGGAAGCCAAAGC3′
*Tango6*	5′CGTGGTTCATGAGGTGACAG3′	5′GTTGCTTTCTGGCTGAGTCC3
*Coq3*	5′CGGGACCATGTGCTTTAGAT3′	5′ACCTCCCTGCTGTCAACTGT3
*Celf6*	5′CAGCTCTGCCTCAACAACAA3′	5′AGAGACAACAGCTCCGAAGG3
*Casr*	5′TTCCTCCCTGATTGCTATGC3′	5′TTCCTGGGATGGACTTTCTG3
*Mep1a*	5′GCTCTGGGATTCTTCCATGA3′	5′GGTCTGTGATGGTGTTGTCG3
U6	5′CTCGCTTCGGCAGCACA3′	5′AACGCTTCACGAATTTGCGT3′
β-actin	5′CCTGTACGCCAACACAGTGC3′	5′ATACTCCTGCTTGCTGATCC3′

* PCR cassettes used (Qiagen, Valencia, CA, USA) contained reverse primers. Only forward primers are listed.

### Microarrays

DNA microarray analyses were conducted to compare the hippocampal tissues of mice from the POCD and control groups. The Agilent mouse lncRNA + mRNA Array V.1.0 platform was utilized to analyze lncRNA and mRNA profiles, and the Affymetrix _7G_ miRNA 4.0 Array was used to analyze miRNA profiles. Screening of differentially expressed genes from the study was conducted by the CapitalBio Corporation & National Engineering Research Center for Beijing Biochip Technology. The procedure included the following steps: sample RNA extraction; sample RNA quality analysis; total RNA > 1 μg; cDNA formation; sense cDNA fragmentation; biotin labeling; chip hybridization; chip elution; chip scanning; signal value detecting and filtering, and removing signals that were weaker than the background; and hybridization image analysis. The Agilent GeneSpring software package was utilized to normalize and analyze extracted lncRNA and mRNA data, and miRNA data were analyzed using the miRNA QC Tool with Affymetrix default analysis settings and quantile as the normalization method.

### Construction of the ceRNA co-expression network

The ceRNA co-expression network was constructed as follows: (1) negatively correlated miRNA-mRNA pairs were screened, where the mRNA was the target of the miRNA; (2) negatively correlated miRNA-lncRNA pairs were screened, where the lncRNA was the target of the miRNA; (3) a lncRNA-mRNA integration analysis was performed by screening lncRNA-mRNA pairs with positive co-expression relationships; (4) a list of lncRNA-mRNA pairs was constructed based on the shared miRNA by comparing the screened results of (1) and (2); (5) the intersection of the screened results of (3) and (4) was analyzed to identify lncRNA-miRNA-mRNA triplets, also known as ceRNAs.

### Statistical analysis

Data were analyzed using GraphPad PRISM version 6 software (San Diego, CA, USA) and average values in each experimental group were expressed as means ± SD. Student’s *t*-tests were used to analyze behavioral and qRT-PCR data. *P* < 0.05 was considered statistically significant.
